# Respiratory Symptoms and Airway Obstruction in HIV-Infected Subjects in the HAART Era

**DOI:** 10.1371/journal.pone.0006328

**Published:** 2009-07-21

**Authors:** M. Patricia George, Mouhamed Kannass, Laurence Huang, Frank C. Sciurba, Alison Morris

**Affiliations:** 1 Department of Medicine, University of Pittsburgh School of Medicine, Pittsburgh, Pennsylvania, United States of America; 2 Department of Medicine, Division of Pulmonary and Critical Care Medicine, University of Southern California and Will Rogers Pulmonary Research Institute, Los Angeles, California, United States of America; 3 Department of Medicine, San Francisco General Hospital, University of California San Francisco, San Francisco, California, United States of America; McGill University Health Center, Montreal Chest Institute, Canada

## Abstract

**Background:**

Prevalence and risk factors for respiratory symptoms and airway obstruction in HIV-infected subjects in the era of highly active antiretroviral therapy (HAART) are unknown. We evaluated respiratory symptoms and measured airway obstruction to identify the impact of HAART and other risk factors on respiratory symptoms and pulmonary function.

**Methodology/Principal Findings:**

Two hundred thirty-four HIV-infected adults without acute respiratory symptoms were recruited from an HIV clinic. All subjects were interviewed and performed spirometry. Multivariate linear and logistic regressions were performed to determine predictors of respiratory symptoms, forced expiratory volume in one second (FEV_1_) percent predicted, and FEV_1_/forced vital capacity (FEV_1_/FVC). Thirty-one percent of subjects reported at least one respiratory symptom. Smoking status (current or former versus never) (odds ratio [OR] = 2.7, 95% confidence interval [CI] = 1.41–5.22, p = 0.003), higher log plasma HIV viral levels (OR = 1.12, 95%CI = 1.02–1.24, p = 0.02), and lower FEV_1_/FVC (OR = 1.06 for every 0.01 decrease in FEV_1_/FVC, 95%CI = 1.02–1.14, p = 0.001) were independent predictors of respiratory symptoms. Age (p = 0.04), pack-year smoking history (p<0.001), previous bacterial pneumonia (p = 0.007), and HAART use (p = 0.04) were independent predictors of decreased FEV_1_/FVC.

**Conclusions/Significance:**

Respiratory symptoms remain common in HIV-infected subjects, especially in those with a smoking history. Subjects who were older, had a greater pack-year history of smoking, or previous bacterial pneumonia had lower FEV_1_/FVC ratios. Interestingly, use of HAART was independently associated with a decreased FEV_1_/FVC, possibly secondary to an immune response to subclinical infections, increased autoimmunity, or other factors associated with HAART use.

## Introduction

The development of highly active antiretroviral therapy (HAART) has led to impressive declines in morbidity and mortality from the human immunodeficiency virus (HIV) [1]. With decreases in opportunistic infections and malignancies, a new constellation of conditions has emerged that have been linked to antiretroviral medications or to extended periods of time living with HIV. In particular, conditions such as cardiovascular disease, dyslipidemia, insulin resistance, and osteoporosis seem to be accelerated in HIV-infected persons receiving HAART [2], [3]. Immune reconstitution inflammatory syndrome (IRIS) has also been reported in HIV-infected persons initiating HAART who develop inflammatory reactions to either known or occult pathogens, and autoimmune disease has been seen during immune restoration [4], [5].

In the pre-HAART era, HIV-infected persons had a high prevalence of respiratory complaints including cough, shortness of breath, and dyspnea on exertion [6], and HIV infection had been associated with accelerated development of emphysema, airway obstruction, and reduced diffusing capacity for carbon monoxide [7], [8]. Respiratory complaints occurred even in individuals without a history of AIDS or pulmonary infections. Although one study examined chronic obstructive pulmonary disease (COPD) as documented by self-report and ICD-9 diagnoses in HIV-infected patients in the HAART era, no studies have measured airway obstruction directly [9]. Therefore, the impact of HAART on frequency of respiratory symptoms or airway obstruction is unknown.

Risk factors for respiratory symptoms and airway obstruction reported in the pre-HAART era might also have changed with HAART. Most previous studies documented an association between smoking, respiratory symptoms, and airway obstruction among HIV-infected individuals [6], [10]. Other pre-HAART era risk factors for respiratory symptoms included intravenous drug use (IDU), low CD4 cell count, and previous pneumonia [6]. Pre-HAART risk factors for airway obstruction included IDU, previous *Pneumocystis* pneumonia (PCP), and previous bacterial pneumonia [8], [11]. It is important to determine current risk factors for respiratory complaints and airflow obstruction in order to identify and treat affected patients.

We conducted a cross-sectional study of HIV-infected individuals presenting to an HIV clinic for routine care. Our overall aims were to investigate the frequency of respiratory symptoms and airway obstruction during the HAART era, identify risk factors, and explore the relationship of HAART to symptoms and airway obstruction. We report the first prospective study to directly measure pulmonary function in HIV-infected individuals in the HAART era, and the first to report a relationship of HAART and airway obstruction.

## Methods

### Objectives

Our objectives were to determine frequency of respiratory symptoms and airway obstruction during the HAART era, to identify associated risk factors, and to determine the relationship of HAART to symptoms and pulmonary function.

### Participants

Subjects were HIV-infected adult outpatients at the University of Southern California HIV clinic, who were enrolled between September 2003 and September 2004. Subjects who had new or increasing cough, shortness of breath, or fever in the past four weeks were excluded, as were subjects with self-reported asthma.

### Description of Investigations Undertaken

#### Data collection

Data were collected by subject interview and standardized medical record abstraction using pre-determined definitions. Information collected from subjects included age, gender, race and ethnicity, HIV risk factor, medication use, smoking history, and history of an AIDS-defining diagnosis. History of pneumonia was obtained from both patient interview and chart review. HAART was defined as use of at least three medications from two classes of antiretroviral drugs within the previous 3 months as determined by medical record review. Current smokers were defined as those smoking at least one cigarette each day and at least a lifetime total of 100 cigarettes. Former smokers had quit for greater than one year. Subjects were asked about presence or absence of chronic respiratory symptoms including cough, shortness of breath, and dyspnea on exertion. Laboratory values for CD4 cell counts and plasma HIV viral levels were obtained within six months of spirometry testing.

#### Spirometry

All subjects performed spirometry according to American Thoracic Society guidelines [12]. Predicted values were calculated using published reference values [13].

### Ethics

The University of Southern California Institutional Review Board approved the study. All subjects gave written informed consent.

### Statistical Methods

#### Statistical analysis

Data were double-entered to ensure accuracy. Stata 8 (Stata Corporation, College Park, TX) was used for analysis, and significance determined for a p-value ≤0.05. Variables were described using either t-tests and Wilcoxon rank-sum, or chi-square and Fisher's exact.

Percentage of subjects reporting respiratory symptoms was determined for individual symptoms and for any symptom. Univariate analyses were performed to determine clinical variables related to reporting any respiratory symptom. Step-wise forward and backward multivariate logistic regression was performed to determine independent predictors of respiratory symptoms by including variables significant at a level of p≤0.1 in univariate analyses. If two variables were significantly correlated (i.e., smoking status and pack-year history), the variable with the strongest univariate relationship to the outcome was included. Interactions of variables were explored. Model fit was assessed using the Hosmer-Lemeshow goodness-of-fit test [14].

Forced expiratory volume in one second (FEV_1_) percent predicted, forced vital capacity (FVC) percent predicted, and FEV_1_/FVC were described for the entire cohort. The percentages of subjects with airway obstruction as determined both by FEV_1_/FVC below the 5% lower limit of normal adjusted for age and FEV_1_/FVC less than 0.70 were calculated [12]. Subjects with airway obstruction were also categorized according to the Global Initiative on Chronic Obstructive Lung Disease (GOLD) staging [15]. Univariate analyses were performed to determine variables associated with FEV_1_ percent predicted and FEV_1_/FVC. Stepwise forward and backward multivariate linear regression was performed to determine independent predictors of FEV_1_/FVC by including variables at a level of p≤0.1 in univariate analyses. The strongest univariate predictor was included in the multivariate model if two variables were significantly correlated. CD4 cell counts and plasma HIV viral levels were missing for 13 subjects. We compared models with and without subjects with missing data, and there were no significant differences. Data on HAART use were missing or incomplete (i.e. subjects reported recent use of fewer than 3 medications) in 19 subjects. Models were run without subjects who lacked adequate information on HAART use. For subjects reporting use of less than three antiretrovirals, we ran models including them in the HAART group and excluding them as well. There were no significant differences between these two models and because we felt the data were less reliable in the subjects reporting non-standard ART regimens, we chose to exclude them in the final model. We explored interactions between model variables. Because we were specifically interested in the relationship of HAART to FEV_1_/FVC, we investigated other potential interactions and modifying effects for relationship of HAART use to FEV_1_/FVC. To account for confounding, we explored potential mediators and moderators, such as plasma HIV viral levels, CD4 cell counts, and smoking history, using linear regression as described [16]. Normality of model residuals was assessed.

## Results

### Clinical characteristics

Two hundred and thirty-four subjects participated. The majority (82.5%) were men (Table 1). Subjects ranged in age from 26 years to 70 years, with a mean age of 44.1 years. There was a high prevalence of smoking as 59.8% were either current or former smokers. Among smokers, the median number of pack-years was 10. The median CD4 cell count in the cohort was 371 cells/μl and the majority (83.3%) of subjects were receiving HAART. HAART users were more likely to be male (odds ratio [OR] = 4.16, 95% confidence interval [CI] = 1.56-11.11, p = 0.007) and less likely to be current or former smokers (OR = 0.32, 95%CI = 0.08–0.92, p = 0.004) (Table 2). Subjects on HAART had lower log plasma HIV viral levels (6.4 copies/ml vs. 8.0 copies/ml, p = 0.02), but lower CD4 cell counts (333 cells/μl vs. 554 cells/μl, p = 0.002). There was no significant difference in time HIV-infected between HAART users and those not using HAART (8 years vs. 10 years, p = 0.6).

**Table 1:** Characteristics of HIV-infected subjects according to respiratory symptoms.

Characteristic	Entire cohort	No symptoms	Symptoms	*P*	OR (95% CI)
	n = 234	n = 161	n = 73		
Age, mean years (SD)	44.1 (9.4)	43.1 (9.9)	46.4 (8.0)	0.002	1.04 (1.01–1.07)*
Gender					
Male, n (%)	193 (82.5)	134 (83.2)	59 (80.8)	0.65	
Female, n (%)	41 (17.5)	27 (16.8)	14 (19.2)		
Race/ethnicity					
White, n (%)	32 (13.7)	18 (11.2)	14 (19.2)	0.40	
African-American, n (%)	66 (28.2)	43 (26.7)	23 (31.5)		
Hispanic, n (%)	124 (53.0)	92 (57.1)	32 (43.8)		
Other, n (%)	12 (5.1)	8 (5.0)	4 (5.5)		
HIV risk factor (n = 223)					
MSM, n (%)	107 (48.0)	74 (49.0)	33 (45.8)	0.93	
IDU, n (%)	27 (12.1)	14 (9.3)	13 (18.1)	0.04	2.30 (1.01–5.13)
Heterosexual, n (%)	89 (39.9)	63 (41.7)	26 (36.1)	0.61	
Smoking history¶					
Current, n (%)	87 (37.1)	51 (31.7)	36 (49.3)	0.0003	3.45 (1.64–7.14)
Former, n (%)	53 (22.6)	32 (19.9)	21 (28.8)	<0.001	4.76 (2.13–10.00)
Never, n (%)	94 (40.2)	78 (48.4)	16 (21.9)		
Pack-year history, median years, (range)	1.1 (0–90)	0.5 (0–90)	4.5 (0–60)	0.04	1.02 (1.001–1.05)*
CD4 cell count, median cells/μl (range)	371 (3–1368)	380 (14–1010)	313 (3–1368)	0.86	
Log plasma HIV viral level, mean copies/ml (SD)	6.5 (2.8)	6.2 (2.7)	7.2 (3.1)	0.02	1.12 (1.02–1.24)
History of AIDS, n (%)	150 (64.1)	102 (63.4)	48 (65.8)	0.72	
Use of HAART, n (%)	195 (83.3)	133 (82.6)	62 (84.9)	0.86	
History of PCP, n (%)	37 (15.8)	25 (15.5)	12 (16.4)	0.86	
History of BP, n (%)	64 (27.4)	37 (23.0)	27 (37.0)	0.02	2.01 (1.10–3.67)
Hepatitis C, n (%)	29 (12.4)	16 (9.9)	13 (17.8)	0.09	
FEV_1_ percent predicted, mean (SD)	99.1 (15.7)	100.0 (15.2)	96.9 (16.6)	0.15	
FEV_1_/FVC, mean (SD)	0.82 (0.07)	0.81 (0.07)	0.78 (0.07)	0.001	1.06 (1.02–1.14) ^

Note: BP = bacterial pneumonia; CI = confidence interval; HAART = highly active antiretroviral therapy; IDU = intravenous drug use; MSM = men who have sex with men; OR = odds ratio; PCP = *Pneumocystis* pneumonia; SD = standard deviation.

* per year.

^per 0.01 decrease.

¶ Odds ratio as compared to never smokers.

**Table 2:** Characteristics of HIV-infected subjects according to use of HAART.

Characteristic	HAART (n = 195)	Non-HAART (n = 20)	*P*
Age, mean years (SD)	44.4 (9.3)	42.0 (11.0)	0.28
Gender			
Male, n (%)	168 (86.2)	12 (60.0)	0.007
Female, n (%)	27 (13.9)	8 (40.0)	
Race/ethnicity			
White, n (%)	28 (14.4)	3 (15.0)	0.03
African-American, n (%)	54 (27.7)	11 (55.0)	
Hispanic, n (%)	106 (54.4)	5 (25.0)	
Other, n (%)	7 (3.6)	1 (5.0)	
HIV risk factor (n = 187)			
MSM, n (%)	74 (44.3)	6 (30.0)	0.77
IDU, n (%)	22 (13.2)	3 (15.0)	
Heterosexual, n (%)	71 (42.5)	11 (55.0)	
Smoking history			
Current, n (%)	65 (33.3)	14 (70.0)	0.004
Former, n (%)	45 (2.3)	2 (10.0)	
Never, n (%)	85 (43.6)	4 (20.0)	
Pack-year history, median years (range)	0.55 (0–90)	10 (0–37)	0.04
CD4 cell count, median cells/μL (range)	333 (3–1076)	554 (124–1368)	0.002
Log plasma HIV viral level, mean copies/mL (SD)	6.4 (2.9)	8.0 (2.6)	0.02
History of AIDS, n (%)	137 (70.2)	2 (10.0)	<0.001
History of PCP, n (%)	29 (14.9)	1 (5.0)	0.32
History of BP, n (%)	53 (27.2)	8 (40.0)	0.30
Hepatitis C, n (%)	23 (11.8)	4 (20.0)	0.29
Time HIV-infected, median years (range)	8 (1–24)	10 (1–21)	0.60

Note: BP = bacterial pneumonia; CI = confidence interval; HAART = highly active antiretroviral therapy; IDU = intravenous drug use; MSM = men who have sex with men; OR = odds ratio; PCP = *Pneumocystis* pneumonia; SD = standard deviation.

### Respiratory symptoms

**Figure 1:**
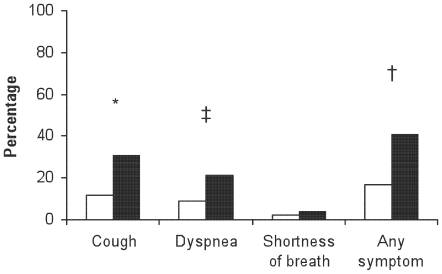
Prevalence of respiratory symptoms in HIV-infected subjects according to smoking history. Current/former smokers shown in black, never smokers in white. Dyspnea = dyspnea on exertion, shortness of breath = shortness of breath at rest. ^*^p = 0.001; ^‡^p = 0.01; ^†^p<0.001.

Prevalence of any respiratory symptom was 31.5%. Cough was most frequently reported, occurring in 23.0% of subjects. Sixteen percent of subjects reported dyspnea on exertion and 3.0% complained of shortness of breath at rest. Current or former smokers were more symptomatic than never smokers (Figure 1).

Age, intravenous drug use, smoking history, higher log plasma HIV viral levels and a history of bacterial pneumonia were associated with a higher likelihood of reporting respiratory symptoms (Table 1). There were no differences in FEV_1_ percent predicted of those with and without symptoms, but FEV_1_/FVC was significantly lower among symptomatic subjects. Multivariate analysis demonstrated that current or former smoking status (OR = 2.7, 95% CI = 1.41–5.22, p = 0.003), higher log serum HIV viral levels (OR = 1.12, 95% CI = 1.02–1.24, p = 0.02), and lower FEV_1_/FVC (OR = 1.06, 95% CI = 1.02–1.14, p = 0.001) were independent predictors of respiratory symptoms.

### Spirometry

Most subjects (93.2%) had normal spirometry. Mean FEV_1_ was 99.1 percent of predicted. Mean FVC was 94.0 percent of predicted, and mean FEV_1_/FVC was 0.82. The prevalence of clinical obstruction as determined by the 5% lower limit of age-adjusted normal was 8.6% and by the FEV_1_/FVC below 0.70 was 6.8%. Of these subjects, 62.5% were classified as GOLD stage I, and 37.5% were GOLD stage II.

No characteristics were significantly associated with FEV_1_ including age, gender, race/ethnicity, HIV risk factor, smoking or pack-year history, CD4 cell count or HIV viral level, use of HAART, or history of pneumonia. In contrast, several clinical characteristics were associated with a lower FEV_1_/FVC (Table 3). The ratio decreased with increasing age (p<0.001) and those who were Hispanic had a higher FEV_1_/FVC than other racial groups (0.81 versus 0.79, p = 0.01). Subjects who were current or former smokers had a lower FEV_1_/FVC than never smokers (0.79 versus 0.82, p = 0.01) and FEV_1_/FVC decreased with increasing pack-year history (p<0.001). Subjects with a history of hepatitis C had a lower ratio than those who did not (0.77 versus 0.81, p = 0.02), as did those who reported a history of bacterial pneumonia (0.78 versus 0.81, p = 0.001). There was a trend for subjects receiving HAART to have a lower FEV_1_/FVC (0.80 versus 0.83, p = 0.09). Multivariate modeling demonstrated that older age (p = 0.04), pack-year history of smoking (p<0.001), and history of bacterial pneumonia (p = 0.007) were independent predictors of lower FEV_1_/FVC (Table 3). Use of HAART was also an independent predictor of a lower FEV_1_/FVC (p = 0.04). There were no significant interactions among variables in the model and no significant mediators or modifiers responsible for the effect of HAART. Sixteen subjects (8.2%) receiving HAART met clinical criteria for airway obstruction (FEV_1_/FVC<0.70), but none of the subjects not receiving HAART had clinical obstruction (p = 0.37). There were no relationships of FEV_1_/FVC to particular classes of antiretroviral therapy, although we were unable to assess nucleoside reverse transcriptase inhibitors (NRTI) separately from HAART as virtually all subjects receiving HAART were using an NRTI.

**Table 3:** Predictors of airway obstruction in HIV-infected subjects.

**Univariate predictors**	**Coefficient**	**P**
Age	−0.18	<0.001
Hispanic ethnicity (versus non-Hispanic)	2.3	0.01
Smoking history (former/current versus never)	−2.2	0.01
Smoking pack-year history	−0.19	<0.001
Hepatitis C	−3.2	0.02
History of bacteria pneumonia	−3.2	0.001
Use of HAART	−2.8	0.09
**Multivariate predictors**		
Age	−0.10	0.04
Smoking pack-year history	−0.15	<0.001
History of bacterial pneumonia	−2.8	0.007
Use of HAART	−3.2	0.04

Note: HAART = highly active antiretroviral therapy.

## Discussion

This study is the first to prospectively examine respiratory symptoms and directly measure spirometry in the HIV-infected population in the era of HAART. It is also the first study to report an association between HAART use and airway obstruction. Respiratory symptoms remain common in the current era and are associated with age, smoking history, lower FEV_1_/FVC, and higher HIV viral levels. We found that a lower FEV_1_/FVC ratio is independently related to age, pack-year smoking history, and history of bacterial pneumonia. Interestingly, we also discovered an independent relationship of HAART use with lower FEV_1_/FVC.

Previous studies documented a high prevalence of respiratory symptoms in the pre-HAART era [6], [17]. Diaz found that approximately 42% of 327 HIV-infected individuals reported dyspnea and 40% reported cough [6]. In the current cohort, only 16% complained of dyspnea and 23% of cough. Overall, about one-third of our subjects had respiratory symptoms. Although it is difficult to compare cohorts, respiratory symptoms may be somewhat less prevalent than previous, although still fairly common. Risk factors for respiratory symptoms were similar in both studies. Diaz reported that cigarette smoking, IDU, low CD4 cell count, and previous history of pneumonia were associated with increased respiratory symptoms. Similarly, we found that smoking was the strongest predictor of respiratory symptoms and that age, IDU, and history of bacterial pneumonia were associated with increased respiratory symptoms. We did not find that CD4 cell count was associated with respiratory symptoms, but high plasma HIV viral levels were. Also, subjects with respiratory symptoms had lower FEV_1_/FVC values than those not reporting symptoms, suggesting that symptoms might be the result of airway obstruction. Combined, these studies argue for increased efforts at smoking cessation, substance abuse intervention, and routine influenza and pneumococcal vaccinations in an effort to decrease respiratory symptoms in HIV-infected patients.

Many studies from the pre-HAART era demonstrated that HIV is associated with airway obstruction, emphysema, and decreased diffusing capacity for carbon monoxide [7], [8], [11], [18]. We found that clinical airway obstruction was present in 6.8 percent of subjects; however, given the different cohorts in these various studies, it is challenging to make direct pre-HAART and post-HAART prevalence comparisons. A recent study of HIV-infected veterans in the HAART era reported that approximately 10 percent carried a diagnosis of COPD based on ICD-9 codes [19], but direct measurements of spirometry have not previously been performed in subjects receiving HAART. Risk factors for decreased FEV_1_/FVC in our cohort were generally consistent with pre-HAART risk factors and included age, smoking pack-year history, and prior bacterial pneumonia [7], [11]. Although a history of PCP has also been associated with obstruction [11], we did not find this relationship, possibly secondary to lack of power or inaccuracy in diagnosis of PCP.

Most notably, we found that HAART use was associated with a decreased FEV_1_/FVC after controlling for other independent risk factors such as age, cigarette smoking, intravenous drug use, and previous pneumonia. Although absolute differences in FEV_1_/FVC were small in subjects receiving HAART, these subjects were young and face a lifetime of antiretroviral treatment. Therefore, they may be at risk of developing clinically significant disease as they age.

Airway obstruction may be the latest in a series of chronic conditions linked to HAART. Prolonged HIV infection and HAART have been associated with cardiovascular disease, metabolic syndrome, osteoporosis, rheumatologic disorders, and thyroid disease [20], [21]. The mechanistic link between HAART and airway obstruction is not known, but potential explanations include direct effects of HAART in the lung, restoration of the immune system allowing for renewed response to subclinical infections, and/or the development of autoimmunity.

HAART-associated cardiovascular disease, metabolic syndrome, and osteoporosis may be directly related to particular antiretroviral agents, particularly protease inhibitors [22], [23], but there are no published reports of HAART medications linked to airway obstruction. We did not see a statistically significant association between protease inhibitors and obstruction, but we might have lacked sufficient power to detect this relationship. Further research is warranted to investigate the direct pulmonary effects of HAART.

HAART might also potentiate airway obstruction through indirect mechanisms such as aberrant immune restoration. IRIS is a well-documented side-effect of HAART initiation that occurs in response to known or occult pathogens [4]. Individuals placed on HAART may experience symptoms such as fever, worsening lung infiltrates, and lymphadenopathy [4], [24]. These symptoms likely result from release of memory CD4+ T-cells occurring weeks to months after HAART initiation. We propose that there may be a “modified” IRIS that, while not resulting in overt respiratory disease, stimulates pulmonary inflammation in response to occult or colonizing pathogens and subsequently results in airway obstruction.

In the non-HIV-infected population, the vicious circle hypothesis has been proposed as one mechanism leading to COPD [25]. This theory postulates that continued inflammation in response to microorganisms perpetuates lung damage and COPD. Bacterial pathogens such as *Streptococcus pneumonia* and *Haemophilus influenzae*, and viral pathogens such as adenovirus, have been implicated in COPD pathogenesis [26], [27]. *Pneumocystis jirovecii* colonization has also been linked to COPD in the non-HIV-infected population and has been found using polymerase chain reaction in as many as 70 percent of HIV-infected individuals without acute *Pneumocystis* pneumonia [28], [29]. One of these organisms or HIV itself could act, with a persistent, but low organism burden, as a nidus of chronic inflammation in the context of improved immune function after starting HAART.

Another possible explanation of the association of HAART and airway obstruction, which does not necessarily exclude a role for infections, is that an autoimmune response develops after initiating HAART. Organ-specific autoimmunity has been demonstrated as a complication of HAART and occurs later than infection-associated IRIS [5], [30]. Conditions such as autoimmune thyroid disease, sarcoidosis, autoimmune hepatitis, and arthritis have been noted after beginning HAART [20], [21], [30], [31], [32]. Detectable anti-thyroid antibodies appear after HAART initiation, and clinical thyroid disease is associated with a lower pre-HAART CD4 cell count [20], [21]. The proposed mechanism of autoimmunity during immune restoration involves release of naïve T-cells and generally occurs more than six months after HAART initiation. Autoreactive T-cells are subsequently more likely to be activated in the setting of infections, thymic dysfunction, or altered cytokine profiles associated with HIV infection and HAART [20], [33], [34], [35]. It has also been postulated that production of T-regulatory cells is impaired in HIV-infected subjects during HAART and might lead to decreased ability to suppress autoimmunity [20], [36]. Others have shown that numbers of mucosal T-regulatory cells decrease with HAART initiation [37], and it is possible that similar changes occur in the lungs resulting in decreased ability to suppress the autoimmune response.

Autoimmunity, either in response to infectious agents or other triggers, has been linked to COPD in the non-HIV-infected population [38], [39], [40]. Agusti proposed that an acquired immune response to self-epitopes was an important contributor to the inflammation and pulmonary damage seen in COPD [38]. Recent studies have shown that non-HIV-infected COPD patients have increased anti-elastin and anti-epithelial antibodies [39], [40]. The susceptibility of HIV-infected patients on HAART to developing autoimmunity in the setting of immune restoration, combined with the findings supporting an autoimmune pathogenesis in COPD, indicate that autoimmune mechanisms may also be involved in HIV-related COPD.

### Limitations

There are several limitations of our study. First, it was a cross-sectional observational study of HIV-infected individuals; thus limiting our ability to determine causation. Use of chart review for assessing certain clinical variables is also difficult. We were unable to assess effects of HAART on progression of airway obstruction or to determine effects of length of HAART use, HAART compliance, or nadir CD4 cell count, as this information was not reliably available. Use of plasma HIV viral levels as surrogate markers of adherence is also difficult, but HAART users did have lower HIV viral levels than those not receiving HAART, suggesting that at least some subjects were compliant with HAART. It also did not appear that length of HIV infection was associated with airway obstruction, but this information is difficult to obtain accurately. In addition, pulmonary testing was limited to pre-bronchodilator spirometry. Subjects with a history of asthma were excluded, but we cannot rule out the possibility that some subjects had asthma. Airway hyperresponsiveness was previously reported in HIV-infected subjects, although not at a significantly higher prevalence than in controls and it was more common in those with a self-reported history of asthma, a group excluded from our study [41]. We were not able to examine the association of HAART and diffusing capacity for carbon monoxide or anatomic emphysema as demonstrated on computed tomography. Thus, we might have underestimated the association of HAART and pulmonary disease. Nonetheless, the finding that HAART is associated with airway obstruction is important whether it represents asthma or COPD, and future studies are needed to determine the exact phenotype associated with HIV and HAART use. Finally, there might be unmeasured variables associated with HAART use and airway obstruction that were not considered in our model; however, we included most previously reported variables associated with obstruction and, in fact, HAART users were actually less likely to smoke, yet had more obstruction.

As this study was performed at a single center, results may not be able to be generalized to all populations of HIV-infected patients. Subjects were from an urban area, and populations in different settings or different geographic locations might not be similar to the one reported here. Inclusion of relatively healthy subjects who attended an outpatient clinic may have also biased results in finding a lower prevalence of respiratory symptoms and pulmonary function deficits than would be found in a cohort with more advanced disease or in those who do not seek primary care.

This study is the first prospective examination of airway obstruction and risk factors in HIV-infected persons during the HAART era. While most risk factors for airway obstruction were similar to those seen in the pre-HAART era, we found a novel, independent association of HAART and airway obstruction. Although the effect of HAART was small, the subjects in our study were young and the mild changes identified here could become clinically important over a lifetime of antiretroviral therapy. Future studies are needed to improve understanding of the relationship of HAART and pulmonary abnormalities, to determine progression of abnormalities over time, and to discover the mechanism of this association in order to provide treatment or prevention.
